# Fractal analysis of the structural complexity of the connective tissue in human carotid bodies

**DOI:** 10.3389/fphys.2014.00432

**Published:** 2014-11-05

**Authors:** Diego Guidolin, Andrea Porzionato, Cinzia Tortorella, Veronica Macchi, Raffaele De Caro

**Affiliations:** Section of Human Anatomy, Department of Molecular Medicine, University of PadovaPadova, Italy

**Keywords:** morphometry, fractal parameters, co-occurrence matrix, Morisita's index, carotid body, drug-related death, heroin, aging

## Abstract

The carotid body (CB) may undergo different structural changes during perinatal development, aging, or in response to environmental stimuli. In the previous literature, morphometric approaches to evaluate these changes have considered quantitative first order parameters, such as volumes or densities, while changes in spatial disposition and/or complexity of structural components have not yet been considered. In the present study, different strategies for addressing morphological complexity of CB, apart from the overall amount of each tissue component, were evaluated and compared. In particular, we considered the spatial distribution of connective tissue in the carotid bodies of young control subjects, young opiate-related deaths and aged subjects, through analysis of dispersion (Morisita's index), gray level co-occurrence matrix (entropy, angular second moment, variance, correlation), and fractal analysis (fractal dimension, lacunarity). Opiate-related deaths and aged subjects showed a comparable increase in connective tissue with respect to young controls. However, the Morisita's index (*p* < 0.05), angular second moment (*p* < 0.05), fractal dimension (*p* < 0.01), and lacunarity (*p* < 0.01) permitted to identify significant differences in the disposition of the connective tissue between these two series. A receiver operating characteristic (ROC) curve was also calculated to evaluate the efficiency of each parameter. The fractal dimension and lacunarity, with areas under the ROC curve of 0.9651 (excellent accuracy) and 0.8835 (good accuracy), respectively, showed the highest discriminatory power. They evidenced higher level of structural complexity in the carotid bodies of opiate-related deaths than old controls, due to more complex branching of intralobular connective tissue. Further analyses will have to consider the suitability of these approaches to address other morphological features of the CB, such as different cell populations, vascularization, and innervation.

## Introduction

The carotid body (CB) is the main peripheral arterial chemoreceptor, sensitive to reduction in pO_2_ and pH and to increases in pCO_2_. From a structural point of view, it is composed of lobules containing type I cells, positive for tyrosine hydroxylase, and type II cells, positive for glial fibrillary acidic protein. Type I cells are considered the true chemoreceptor elements. They are roundish and produce many different neurotransmitters and peptide neuromodulators. Type II cells are fusiform and envelop clusters of type I cells. They are usually considered supportive cells, although they may also be stem cell precursors for type I cells (Pardal et al., 2007; Platero-Luengo et al., 2014) and probably co-ordinate chemosensory transduction through interactions with the other cells of the CB (Tse et al., 2012). Connective tissue also characterizes the CB structure, mainly delimiting the glomic lobules (interlobular connective tissue) and partly branching in the lobular context (intralobular connective tissue). Neurotransmitters and neuromodulators released by type I cells mainly act on the afferent endings of the carotid sinus nerve, arising from the glossopharyngeal nerve. The CB also shows sensory innervation from jugular and nodose ganglia, post-ganglionic sympathetic nerve fibers from the superior cervical ganglion, and preganglionic parasympathetic and sympathetic fibers reaching local ganglion cells. Moreover, the CB is the structure in the body with the highest blood flow (Daly et al., 1954; Barnett et al., 1988) and local changes in blood flow have been considered to be involved in CB chemoreceptor discharge (Joels and Neil, 1963; Kirby and McQueen, 1984; Porzionato et al., 2006, 2011a,b).

The CB undergoes structural and functional changes during perinatal development (e.g., Porzionato et al., 2008a,b; De Caro et al., 2013), aging (e.g., Di Giulio et al., 2009, 2012; Zara et al., 2013a) and in response to a variety of environmental stimuli, such as chronic sustained hypoxia (e.g., Pardal et al., 2007; Platero-Luengo et al., 2014), chronic intermittent hypoxia (e.g., Iturriaga et al., 2009), chronic hyperoxia (e.g., Bavis et al., 2013), and exposure to nicotine (e.g., Stéphan-Blanchard et al., 2013). Several morphometrical approaches have been involved to address structural changes in the CB. Most morphometrical parameters addressed in the literature are first order parameters, such as volumes or densities. Volume analyses may involve CB *in toto* or its different components (parenchyma, interlobular or intralobular connective tissue, vessels) (e.g., Dinsdale et al., 1977; Lack et al., 1986; Clarke et al., 2000; Porzionato et al., 2005). Innervation of the CB has mainly been evaluated in terms of density values (e.g., Kusakabe et al., 2003, 2004). The different cell types of the CB (type I and II cells, progenitors, macrophages, mast cells, and other immune cells) have been considered in the literature in terms of cell densities or total cell numbers (e.g., Pardal et al., 2007; Porzionato et al., 2013). Computer-assisted image analysis of protein expression in immunostained sections has also been performed through quantification of the immunoreactive area in order to estimate the percentage of tissue exhibiting positivity (e.g., Di Giulio et al., 2012; Zara et al., 2013b).

Size parameters alone, however, may be inadequate to fully characterize the microarchitecture generated by tissue components such as connective tissue, type II cells, vessels, and innervation, all characterized by a quite complex spatial arrangement. In fact, a pattern of this type, for each given size, can generate in the available space substantially different spatial textures characterized by different degrees of homogeneity and morphological complexity (Guidolin et al., 2004a,b). In the present study possible strategies to morphometrically estimate these morphological features of the CB tissue have been considered. In particular, they will be used for the analysis of the pattern of fibrosis induced in the CB by normal aging and opiate abuse in young people. Since data exist showing that a comparable increase in the amount of connective tissue in the CB occurs in both conditions, but with a likely different pattern of spatial distribution (Porzionato et al., 2005), this specific example can allow a test of the efficiency of the considered methods.

## Materials and methods

### Tissue samples

Materials consisted of carotid bodies obtained at autopsy from 35 subjects who died of heroin/morphine intoxication (26 males, nine females; mean age (± *SD*) 26 ± 3.5 years). In all cases, there was a clinical history of at least 3 years of heroin addiction. The other two groups for comparison consisted of 10 young (five males, five females; mean age 22 ± 3.4 years) and 10 aged subjects (five males, five females; mean age 66.5 ± 3.5 years) who died of trauma. All subjects were clinically without chronic pulmonary or cardiovascular disease. Cardiac hypertrophy or preceding myocardial infarction were excluded at autopsy. Autopsies were performed between 24 and 78 h after death. Specimens were taken of the right carotid bifurcation, including 20 mm of the common carotid and 20 mm of the internal and external carotid arteries.

Autopsies and all the procedures applied to process samples from human tissues have been performed according to the Italian Mortuary Police Legislation.

### Histological techniques

Tissues were fixed in 10% phosphate-buffered formalin for 72 h, dehydrated through ascending alcohols and xylene, and paraffin embedded. Longitudinal serial sections, 5 μm thick, of the whole carotid bifurcation were then obtained, de-waxed (xylene and alcohol progressively at lower concentrations), and stained with Azan-Mallory (AM).

### Image analysis procedures

All the image analysis procedures were performed by using the ImageJ software (Schneider et al., 2012), freely available at http://rsb.info.nih.gov/ij/. They can be summarized as follows.

#### Image acquisition and pre-processing

**Figure 1:**
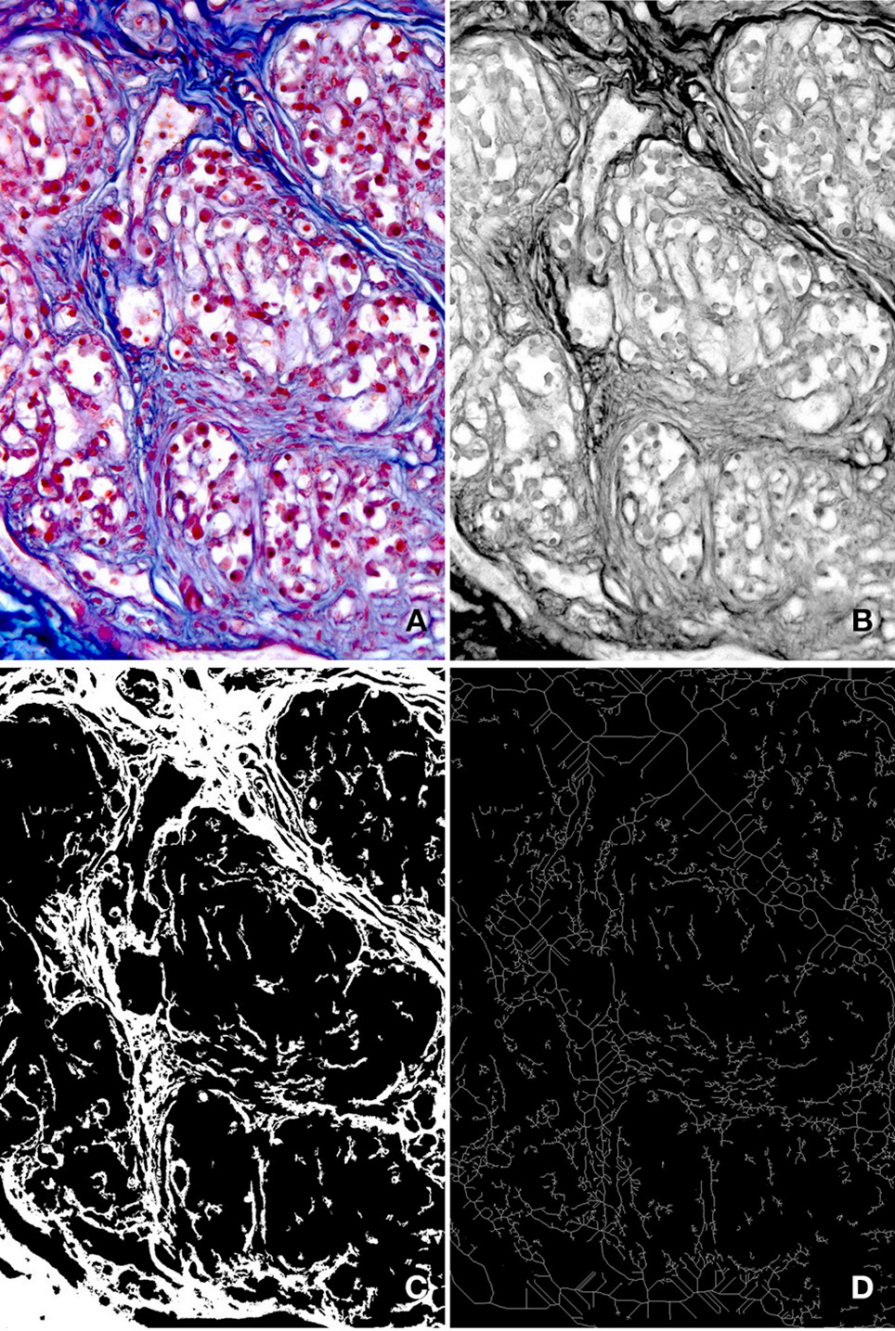
**Main steps of the image analysis procedure. (A)** Full color (RGB, 24-bit) digital image of a microscope field stained with AM (primary magnification ×20). **(B)** Gray level image corresponding to the red component of the image in **(A)**. Due to the high contrast between connective tissue and parenchyma it exhibits, it was used to estimate the GLCM and to discriminate the connective tissue by proper thresholding. **(C)** Binary image of the connective tissue pattern, used to estimate the percent area it occupies and the Morisita's index. **(D)** Binary skeleton of the image in **(C)**, from which fractal parameters were estimated.

Bright-field images of the AM-stained preparations were acquired by using a Leica DMR microscope (Leica Microsystems, Wetzlar, Germany) and a high resolution digital camera (DC 200, Leica Microsystems). At a primary magnification of ×20 one field per section, randomly chosen within the CB tissue, was selected and its image acquired in full colors (RGB, 24-bit), processed to correct shading, then filed TIFF (Figure 1A).

Since it showed the best contrast between the connective tissue and the CB parenchima, the red component of each acquired RGB image was selected for further processing (Figure 1B). Stromal structures and filaments can be easily segmented with conventional thresholding methods, and small remaining artifacts can be removed from the resulting binary image by applying a geometric filter to eliminate profiles within a specified range of area and/or shape (see Russ, 2011), leading to the generation of binary images (Figure 1C) of the connective component.

The amount of CB tissue it accounts for can be directly estimated from the corresponding binary image by evaluating the area fraction occupied by the binary pattern (Russ and Dehoff, 2000).

#### Morphological complexity of the pattern of the connective tissue

The obtained gray-level and binary images illustrated in Figure 1, however, can also represent the input data for procedures aimed at estimating indices able to capture more detailed morphological features, such as a characterization of the overall shape of the patterns generated by connective tissue, and of the way it arranges itself in the tissue. In these respect, three methods were considered in the present study. They are briefly detailed in the sections that follow.

***Analysis of dispersion***. To provide a quantitative evaluation of the dispersion in the tissue space of the binary pattern corresponding to connective tissue, Morisita's index (Morisita, 1962), one of the most robust distribution measures (Myers, 1978), was estimated. For this purpose, the binary image was divided into 12 sub-images and the number of pattern pixels in each sub-picture was evaluated. The index of dispersal (*I_d_*) was then calculated using

$$I_{d}=n\left(\frac{\sum_{i=1}^{n}X_{i}^{2}-N}{N(N-1)}\right)$$

where *n* is the number of sub-images, whereas *N* and *X_i_* represent the number of pattern pixels in the image, and in each sub-image, respectively. The index value increases with increasing spatial dispersion of the pattern.

***Gray level co-occurrence matrix analysis***. Gray level co-occurrence matrix (GLCM) is a fast mathematical method for assessing image structural properties such as homogeneity, complexity, and level of disorder (see Pantic et al., 2012). It was first introduced by Haralick et al. (1973) and is based on a quantitation of the relationship between pixel brightness values in an image. This information can be extracted from a matrix *P*_θ *d*_(*i,j*) (GLCM) describing how frequently two pixels with gray level *i* and *j* appear in the image separated by a distance *d* in the direction θ (Aggarwal and Agrawal, 2012). Haralick et al. (1973) described 14 parameters that can be calculated from the GLCM with the intent of describing the texture of an image. Today, however, those proven as the most useful in experimental and clinical medicine applications (see Losa and Castelli, 2005; Alvarenga et al., 2010; Pantic et al., 2012) are the following ones:

$$\begin{array}{l} \;Entropy=-\sum_{i}\sum_{j}P(i,j)\text{log}(P(i,j))\\ Angular\;second\;moment=\sum_{i}\sum_{j}{\left[P(i,j)\right]}^{2}\\ \;Variance=\sum_{i}\sum_{j}{\left(1-\mu \right)}^{2}P(i,j)\\ \;Correlation=\frac{\sum_{i}\sum_{j}ijP(i,j)-\mu_{x}\mu_{y}}{\sigma_{x}\sigma_{y}} \end{array}$$

where *i* and *j* are coordinates of the co-occurrence matrix, σ′s and μ′s represent means and standard deviations along rows and columns of the matrix. They were computed with ImageJ by using the “texture analysis” plugin, developed by Julio E. Cabrera and Toby C. Cornish, and freely available at http://rsbweb.nih.gov/ij/plugins/texture.html.

***Fractal analysis***. To globally describe the complexity of form in quantitative terms the “Fractal dimension” (D) can be a valuable parameter (Guidolin et al., 2004b). It measures the rate of addition of structural detail with increasing magnification, scale, or resolution (Cutting and Garvin, 1987). D of the binary skeleton (Figure 1D) was estimated using the “box counting” method at multiple origins as indicated by Smith et al. (1996). Briefly, from grids of increasing size overlying the image, the number of boxes containing any pixel was counted. This number was recorded as a function of grid size and D was calculated, as −1 times the slope of the regression line, from a plot of the log of size on the x-axis and the log of box count on the y-axis. To minimize grid location effects, the algorithm started from a number (10 in our case) of locations, generating a set of values for D. The average value over all locations was considered as the final estimate of D. During the same analytical process “Lacunarity” was also calculated. This parameter is a measure of the nonuniformity (heterogeneity) of structure or the degree of structural variance within an object (Smith et al., 1996). It was estimated as the average of the coefficient of variation for pixel density over all grid sizes and locations (Bassinghtwaighte et al., 1994).

To perform the abovementioned analysis, the “FracLac for ImageJ” plugin by Audrey Karperien was used (freely available at http://rsb.info.nih.gov/ij/plugins/fraclac/fraclac.html).

#### Statistical analysis

Statistical analysis was done using GraphPad Prism software (GraphPad Inc., La Jolla, CA, USA) and SPSS statistical package (v. 13.0; IBM, Armonk, NY, USA). Data were analyzed by One-Way analysis of variance followed by Dunnet's test for multiple comparisons vs. the young control group. Bonferroni's test for comparisons between selected groups was used to determine possible statistically significant differences between opiate-addicted and aged cases. *p* < 0.05 was always used as the limit for statistical significance. In addition to the standard statistical difference tests, the ability of the various parameters to discriminate between aging and opiate addiction was estimated by calculating the ROC curves (Metz, 1978). ROC curve is constructed based on the fraction of true positives out of the positives (sensitivity) and the fraction of false positives out of the negatives (specificity), taking into account different thresholds. The potential discriminatory performance of each parameter can be estimated by analyzing the area under its ROC curve (see Zweig and Campbell, 1993). A parameter with no discriminative value would have the area under the ROC curve approximately higher than 0.5 and lower than 0.6. Area between 0.6 and 0.7 indicates “poor” performance, area between 0.7 and 0.8 “fair” performance, and area between 0.8 and 0.9 “good” performance (Zweig and Campbell, 1993; Sandelowsky et al., 2011). Parameters belonging to the category “excellent” usually have the ROC areas higher than 0.9.

## Results

**Figure 2:**
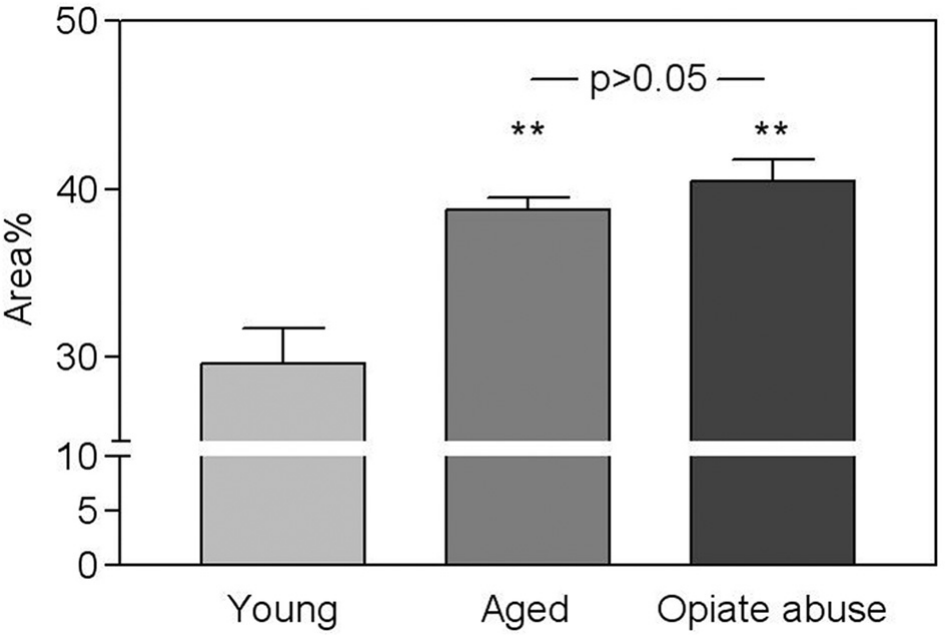
**Fraction of the CB corresponding to connective tissue in young controls, in aged subjects, and in young people who died of chronic opiate abuse**. Values are average percent areas (± s.e.m.). As indicated both aging and opiate abuse induced a significant increase (^**^p < 0.01) of the total amount of connective tissue in the CB with respect to young control subjects. Between the two conditions however, no statistically significant differences can be detected.

**Figure 3:**
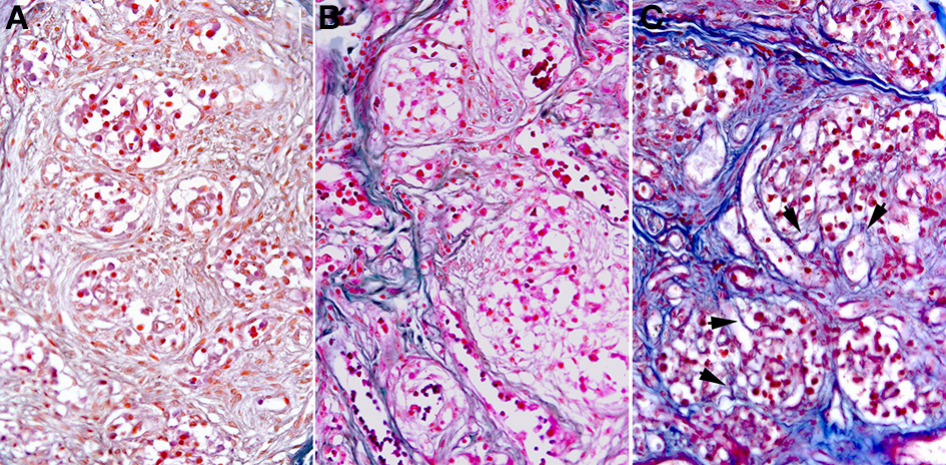
**Microscope fields of AM-stained samples from a young control (A), an aged subject (B), and from a subject who died of chronic opiate abuse (C)**. In the latter the spatial organization of the connective tissue appeared more complex, being characterized by a higher presence of thin branches of connective tissue within the parenchyma lobes. Some of them are highlighted by the arrow heads.

As shown in Figure 2 a similar, statistically significant, increase in the total amount of connective tissue was observed in the CB from aged subjects and from young people who died of opiate intoxication when compared to normal young CB samples. It appeared mainly located between the lobes of parenchyma. From a qualitative point of view, however, some difference between the two groups can be observed in the spatial distribution of this tissue component. In particular a more complex branching of connective tissue within the CB parenchyma lobes seems to characterize the samples from subjects who died of opiate abuse when compared to normal aging (Figure 3).

As shown in Table 1, almost all of the morphometric parameters estimated to characterize the spatial organization of this tissue component indicated significant differences in this tissue feature between young controls and the other two groups of patients, with the only exception of D, that exhibited a similar value in young controls and aged subject and increased significantly only in opiate-addicted group. Interestingly, however, only some of these methods were able discriminate between aged subjects and young people who died of opiate abuse. As summarized in Table 1, “Angular second moment” of the GLCM, fractal parameters (D and lacunarity), and the Morisita's index assumed significantly different values in the two conditions.

**Table 1:** **Mean values (± s.e.m.) of the parameters quantifying textural properties of the connective tissue in CB samples from normal young subjects, from aged subjects, and from young subjects who died of chronic opiate abuse**.

**Feature**	**Young controls**	**Aging**	**Drug-related deaths**
Morisita's index	0.028 ± 0.002	0.060 ± 0.010 (^◦^)	0.083 ± 0.005 (^◦◦,*^)
Angular second moment	0.0048 ± 0.0006	0.0020 ± 0.0007 (^◦◦^)	0.0010 ± 0.0001 (^◦◦,*^)
Variance	151.0 ± 12.9	97.2 ± 4.8 (^◦◦^)	115.4 ± 5.2 (^◦◦^)
Correlation	0.0010 ± 0.00017	0.0004 ± 0.00005 (^◦◦^)	0.0004 ± 0.00003 (^◦◦^)
Entropy	7.91 ± 0.150	8.36 ± 0.099 (^◦^)	8.44 ± 0.079 (^◦◦^)
Fractal dimension	1.43 ± 0.028	1.447 ± 0.013	1.563 ± 0.006 (^◦◦,**^)
Lacunarity	0.828 ± 0.017	0.500 ± 0.021 (^◦◦^)	0.388 ± 0.009 (^◦◦,**^)

◦ *p < 0.05*,

◦◦ p < 0.01 vs. the “Young controls” group;

* *p < 0.05*,

** *p < 0.01 vs. the “Aging” group*.

For each of these parameters a ROC curve was calculated to illustrate the efficiency of the parameter as a binary classifier of CB fibrosis between aged and opiate addicted subjects. As presented in Table 2, the areas under the ROC curves indicated that the fractal parameters (D and lacunarity) were those exhibiting the highest discriminatory power.

**Table 2:** **Area under the ROC curve for the parameters showing any discriminative power between drug-related deaths and aged subjects, and classification of their accuracy (see Zweig and Campbell, 1993; Sandelowsky et al., 2011)**.

**Feature**	**Area under the ROC curve**	**Accuracy**
Morisita's index	0.6529	Poor
Angular second moment	0.7895	Fair
Fractal dimension	0.9651	Excellent
Lacunarity	0.8835	Good

## Discussion

A significant increase in connective tissue and a concomitant reduction in glomic parenchyma are well known characteristics of the CB tissue in both opiate-addicted and aged cases when compared to young controls. Consistently with previously reported data (Porzionato et al., 2005), also in the present study both these conditions led to an increase of the amount of connective tissue from about 30%, that can be observed in normal young people, to more than 40% of the CB. Such a change is in accordance with the findings of Hurst et al. (1985) who found progressive arteriosclerosis of the glomic arteries during aging, to which they ascribed the increase in connective tissue. Also in opiate addicts, the increase in connective tissue may be interpreted as a sign of early tissue aging, that can be ascribed to arteriosclerosis of the glomic arteries, to the recurrent episodes of hypoxia during reaction to opiate assumption, and/or to local inflammatory infiltrates (chronic carotid glomitis) (Porzionato et al., 2009).

The connective tissue, however, exhibits a complex spatial organization. It is mainly formed by large bundles of fibers located between the lobes of parenchyma, from which thin branches of intralobular connective tissue depart. When compared to young controls (see Porzionato et al., 2005), this intralobular component of the pattern appeared increased in both aged and opiate-addicted subjects. An analysis based on the simple estimate of a dimensional parameter (volume fraction) failed in detecting significant differences between the two conditions, although the branching pattern of connective tissue in opiate-addicted subjects appeared (at least qualitatively) more complex.

Thus, in the present study additional morphometric strategies were also explored, based on the analysis of the connective tissue pattern in terms of textural properties (such as homogeneity, complexity and level of disorder) more than overall amount. In particular, three different image analysis methods were tested: analysis of dispersion, analysis of the GLCM, and fractal analysis. Analysis of dispersion has been successfully used to study how a given population or morphological pattern fills the available space (see Goodenough and Goodenough, 2012). GLCM was successfully applied in nuclear magnetic resonance imaging (Li et al., 2009), computed tomography (Huber et al., 2011) and other clinical research areas. In fundamental medical and biology research, it was used to study tissue age-related structural degradation (Shamir et al., 2009), and chromatin structural changes during apoptosis (Losa and Castelli, 2005; Pantic et al., 2012). Fractal analysis proven very useful to characterize the complex morphology of vascular trees and the endothelial cells self-organization *in vitro* (Guidolin et al., 2004a,b). Although the interest was mainly focused on the comparison between opiate-addicted and aged cases (showing comparable amounts of connective tissue), the analysis was also extended to young cases for completeness.

The results indicated that GLCM, Morisita's index, and the fractal parameter “lacunarity” were able to discriminate normal CB tissue samples from those derived from aged or opiate-addicted people. However, since all the above mentioned parameters are measures of *homogeneity* (Morisita, 1962; Smith et al., 1996; Marrón, 2012; Pantic et al., 2013) describing how well a pattern fills the available space as a consequence of the triggering conditions, this finding could be partly related to the increase of connective tissue occurring following aging and opiate addiction.

More interesting findings emerge when conditions characterized by a similar total amount of CB connective tissue were compared. In this respect, the results of the present study indicate that some of the parameters provided by the tested methods (namely angular second moment, lacunarity, and Morisita's index) exhibited discriminatory power between CB normal aging and chronic opiate consumption, although in the presence of comparable amount of connective tissue. These parameters captured the qualitative observation (Porzionato et al., 2005) that following chronic opiate consumption the intralobular connective tissue branches in a more irregular way than in samples from aged subjects.

The parameter D deserves a more specific comment. Unlike the other parameters (estimating spatial homogeneity), it measures the rate of addition of structural detail with increasing magnification, scale, or resolution (Cutting and Garvin, 1987). Thus, it is particularly useful to describe in a compact form the “*complexity of shape*” of a structure. In this respect, the connective tissue pattern in the CB of opiate addicted subjects exhibited a statistically significant higher level of structural complexity when compared to the one observed in aged subjects. Also this finding is consistent with a higher degree of branching likely involving the intralobular regions of the CB. Interestingly, as far as this parameter is concerned, no significant differences were observed between aged and young subjects, showing that this parameter is not influenced (or only minimally) by the overall amount of connective tissue. Instead, the parameter D showed significantly different values between aged and opiate-addicted subjects, confirming the capability of this parameter to catch differences in the complexity of disposition of connective tissue, independently from quantitative aspects. Thus, we may state that beyond differences in the total amount, CB connective tissue is similarly organized in young and aged subjects whereas it is organized in a more complex irregular way in the opiate addicted subjects.

In addition to the standard statistical difference tests, the ability of the abovementioned parameters to discriminate between the two groups with comparable amount of connective tissue was here tested by ROC analysis, a technique essential in clinical sciences for evaluating the potential value of a diagnostic test (Pantic et al., 2013). According to this method, the potentially discriminatory performance of a parameter is evaluated by analyzing the area under the ROC curve. In this study the parameters provided by the fractal analysis (D and lacunarity) showed the highest accuracy and efficiency as binary classifiers. Thus, they appear as a particular useful tool to complement dimensional parameters in order to describe complex tissue patterns (as the one exhibited by the connective tissue in the CB) and the changes they undergo under pathological conditions.

It can also be emphasized that the presented methods are not limited to the specific staining method used in the present study, but they can be applied to all the methods commonly used to visualize the connective tissue (as, for instance, Masson's trichromic and Sirius red), provided the image pre-processing includes a suitable segmentation step for this tissue component, such as color thresholding or color deconvolution (Ruifrok and Johnston, 2001; Rey et al., 2008), which must be considered preliminary to the use of the above methods. Furthermore, the analysis here presented could be easily adapted and extended to provide a morphometric description of spatial complexity of other CB tissue patterns, such as those generated by sustentacular type II cells, vessels, or innervation. In various experimental or clinical conditions, these structural components could have no changes in terms of volume fraction or density but changes in spatial disposition which could have a biological significance and which could be morphometrically described with the above methods, and particularly with fractal analysis.

## Author contributions

All the authors (Diego Guidolin, Andrea Porzionato, Cinzia Tortorella, Veronica Macchi, and Raffaele De Caro) contributed substantially to the conception or design of the work, or to the acquisition, analysis, or interpretation of data of the work. All the authors drafted or revised the work critically for important intellectual content, and they approved the final version to be published. All the authors agree to be accountable for all aspects of the work in ensuring that questions related to the accuracy or integrity of any part of the work are appropriately investigated and resolved.

### Conflict of interest statement

The authors declare that the research was conducted in the absence of any commercial or financial relationships that could be construed as a potential conflict of interest.
